# Comparative Evaluation of Open Versus Closed Rhinoplasty Techniques: A Prospective Clinical Study of Functional and Aesthetic Outcomes

**DOI:** 10.7759/cureus.87253

**Published:** 2025-07-03

**Authors:** Rajshri Yadav, Heena Masrat, Fozia Wani, Nazir A Khan

**Affiliations:** 1 Otorhinolaryngology, Uma Nath Singh Autonomous State Medical College, Jaunpur, IND; 2 Otorhinolaryngology, Urban Primary Health Centre, Srinagar, IND; 3 Otorhinolaryngology, Aga Syed Yousuf Memorial Hospital, Watra Wani, IND; 4 Otorhinolaryngology, Government Medical College, Baramulla, Baramulla, IND

**Keywords:** closed approach, nasal deformity, nose score, open approach, rhinoplasty

## Abstract

Introduction

Rhinoplasty is a widely used procedure aimed at correcting nasal deformities to improve both function and aesthetics. The open and closed techniques are two main surgical approaches, each with specific indications and advantages. This study aimed to compare clinical, functional, and aesthetic outcomes between open and closed rhinoplasty.

Methods

A prospective study was conducted in the Department of Otorhinolaryngology, Sher-I-Kashmir Institute of Medical Sciences, Srinagar, from August 2013 to December 2015. A total of 30 patients with primary external nasal deformities were included, of whom 15 underwent open rhinoplasty and 15 underwent closed rhinoplasty. Pre- and postoperative assessments included photographic analysis of aesthetic angles (nasofrontal, nasofacial, and nasolabial) and the Nasal Obstruction Symptom Evaluation (NOSE) score. Follow-up ranged from 7 to 12 months (mean: 9.5 months).

Results

Of the 30 patients, 17 (56.7%) were male and 13 (43.3%) were female. Post-traumatic deformity was the most common etiology (n = 12; 40%), followed by developmental (n = 10; 33.3%) and congenital causes (n = 7; 23.3%). A total of 21 patients (70%) presented with both cosmetic and functional complaints. Higher aesthetic satisfaction was observed in the open group (12 of 15 patients; 80%) compared to the closed group (9 of 15; 60%). Functional improvement, reflected by reduced NOSE scores, was noted in both groups. Postoperative complications were more frequent in the closed group, including persistent deviation (n = 4; 26.7%) and nasal obstruction (n = 3; 20%).

Conclusion

Both techniques were effective, but open rhinoplasty provided improved aesthetic outcomes and fewer complications, particularly in complex deformities.

## Introduction

The nose, as a central midline structure of the face, plays a pivotal role not only in vital functions such as respiration and olfaction but also in defining facial aesthetics more than any other feature [1]. Any deformity or disproportion in the nose significantly affects facial harmony, often prompting individuals to seek medical or surgical correction [2]. Aesthetic nasal deformities, whether congenital, developmental, or acquired, are among the most common presentations encountered by otorhinolaryngologists, with post-traumatic deformities being particularly prevalent [3].

Rhinoplasty, a surgical intervention designed to reconstruct and reshape the nose, demands a precise understanding of nasal anatomy and its aesthetic subunits and segments. The external nose is topographically divided into nine aesthetic subunits, such as the tip, columella, alar bases, dorsal segments, and alar walls, and six aesthetic segments, including the dorsal nasal segment, lateral nasal walls, and the columellar segment. These divisions aid the surgeon in diagnosing deformities, planning, and executing corrective procedures [4]. Central to nasal aesthetics are three measurable angles: the nasolabial angle, nasofrontal angle, and nasofacial angle. The nasolabial angle, which reflects tip rotation and columellar inclination, typically ranges from 90°-105° in males and 100°-115° in females [5]. The nasofrontal angle, determined between lines extending from the glabella through the nasion and from the nasion to the nasal tip, ranges ideally between 115°-135°, while the nasofacial angle, formed between lines from the glabella to the pogonion and from the nasion to the nasal tip, ranges from 30°-40° [6]. A precise evaluation of these parameters is essential for planning rhinoplasty, as even millimetric changes can substantially influence outcomes.

Rhinoplasty can be classified broadly into surgical (open and closed) and non-surgical approaches. While non-surgical rhinoplasty using fillers offers temporary correction for minor defects, surgical rhinoplasty offers permanent reshaping [7]. The open or external rhinoplasty approach, involving a transcolumellar incision, offers direct visualization of nasal anatomy, allowing accurate diagnosis, structural graft placement, bleeding control, and teaching benefits, making it ideal for complex or revision cases. Conversely, closed or endonasal rhinoplasty avoids external scarring, reduces postoperative swelling, and is technically easier but limits surgical access and precision in complex cases [8]. Techniques such as augmentation rhinoplasty are used to address saddle nose deformity or dorsum depression due to trauma or disease, whereas reduction rhinoplasty addresses dorsal convexity or bulky nasal tips [9].

Revision rhinoplasty is reserved for correcting complications from prior nasal surgeries, such as polybeak deformity or drooping tips. Septoplasty, often performed concurrently, addresses internal deviations contributing to both functional and aesthetic concerns [10]. As Beekhuis famously stated, “As the septum goes, so goes the nose,” emphasizing the interdependence of septal alignment and external nasal shape [11,12]. Ultimately, rhinoplasty is both an art and a science, requiring the surgeon to blend anatomical expertise, aesthetic sensibility, and technical proficiency to achieve optimal results [13]. A comprehensive preoperative assessment, understanding of facial proportions, precise execution, and individualized surgical planning are essential for delivering satisfactory functional and cosmetic outcomes [14]. This study aims to analyze and compare open and closed rhinoplasty techniques, angles of aesthetic importance, and their relevance in surgical planning to contribute toward achieving better postoperative results and patient satisfaction.

## Materials and methods

Study design

This prospective study was carried out in the Postgraduate Department of Otorhinolaryngology, Head and Neck Surgery at Sher-I-Kashmir Institute of Medical Sciences, Srinagar, over a period extending from August 2013 to December 2015. A total of 30 patients, comprising n=17 males and n=13 females, who presented with external nasal deformity and/or septal deviation, were enrolled in this case-control study. The study received approval from the institutional ethics committee and adhered to the principles of the Declaration of Helsinki. All patients were primary cases undergoing rhinoplasty, with n=15 patients managed using the open approach and the remaining n=15 through the closed approach. One patient who had previously undergone septoplasty but presented with persistent external deviation was also included. No revision rhinoplasty cases were included in the study. The duration of follow-up ranged from 7 to 12 months, with a mean follow-up period of 9.5 months.

Inclusion and exclusion criteria

Patients above 15 years and below 55 years of age with external nasal deformity and/or septal deviation were included. Additionally, patients between 10 and 15 years of age with significant nasal deformities and associated septal deviation that adversely affected their quality of life were also considered eligible. Patients with vague or unrealistic expectations, or those seeking rhinoplasty under social pressure, were excluded. Furthermore, patients suffering from debilitating illnesses, chronic granulomatous diseases, or major systemic conditions were not considered for surgery.

Nasal Obstruction Symptom Evaluation (NOSE) score

The NOSE score is interpreted by summing the scores of the five symptom items (each scored from 0 to 4) to obtain a raw total out of 20, which is then multiplied by 5 to convert it into a final score ranging from 0 to 100. Higher scores indicate greater nasal obstruction and more severe symptoms, while lower scores reflect better nasal airflow and patient comfort. A decrease in the NOSE score postoperatively signifies functional improvement and surgical success. Evaluating changes in the NOSE score before and after intervention provides a reliable, patient-reported measure of the functional outcome of rhinoplasty or septoplasty.

Control group

To establish normative data for aesthetic nasal angles in the local population, a control group consisting of n=30 individuals (n=15 males and n=15 females) aged between 15 and 55 years was selected. These individuals were healthy volunteers without any nasal deformity or nasal obstruction. Informed consent was obtained from each participant prior to inclusion in the control group.

Preoperative evaluation

Clinical Examination

All patients underwent a detailed physical and nasal examination. This included inspection to assess the type, site, and extent of the nasal deformity. Standardized preoperative photographs were taken in frontal, right lateral, left lateral, and basal views. Measurements of key aesthetic angles, namely, the nasofrontal, nasofacial, and nasolabial angles, were obtained. The functional assessment included the spatula test and Cottle’s test. Anterior and posterior rhinoscopy were performed, and nasal endoscopy was done when indicated.

Investigations

All patients underwent baseline laboratory investigations, including complete hemogram, blood grouping, liver and kidney function tests, and fasting blood sugar. Chest X-ray (posteroanterior view (PA) view) and ECG were performed for preoperative fitness. Radiographs of the nasal bones and CT scans were obtained where indicated to better define the anatomical deformities.

Preoperative Preparation

Prior to surgery, all patients were admitted and counselled regarding the procedure and its possible outcomes. A detailed written informed consent was obtained. A thorough pre-anaesthetic check-up was conducted. On the day of surgery, patients received IV antibiotics and were kept nil per oral overnight. Pledgets soaked in lignocaine with adrenaline were placed in the nasal cavity 15-20 minutes prior to induction of anaesthesia to reduce intraoperative bleeding.

Intraoperative details

All surgical procedures were performed under general anaesthesia. Hypotensive anaesthesia was preferred in cases managed via the open approach to minimize bleeding and improve visibility. Anaesthesia was induced with propofol and succinylcholine and maintained using oxygen, nitrous oxide, halothane, and atracurium. Intravenous dexamethasone (4-8 mg) was administered to reduce postoperative oedema. The surgical approach (open or closed), the type of incision used, specific techniques employed, and graft materials (only autologous) were documented for each case. The autologous septal cartilage was harvested through a standard hemitransfixion incision during septoplasty, preserving at least 1-1.5 cm of dorsal and caudal strut for structural integrity. In cases where septal cartilage was insufficient or previously harvested, conchal cartilage was obtained from the posterior aspect of the auricle via a postauricular incision, ensuring minimal donor site morbidity. All grafts were shaped intraoperatively according to the structural requirements and secured using suturing techniques appropriate to the site of reconstruction.

Postoperative care

Patients were advised about common postoperative symptoms such as mild nasal bleeding, periorbital pain, oedema, ecchymosis, dry mouth, epiphora, and ear discomfort. Ice-cold sponging and propped-up positioning were recommended to reduce swelling. IV dexamethasone was continued for three days and then tapered off. IV antibiotics and oral analgesics were prescribed for 3-5 days. Decongestant nasal drops were instilled over the nasal packing, which was removed after 48-72 hours. In cases operated by the open approach, an antibiotic ointment was applied to the suture line.

Follow-up

Patients were followed up at one week, three weeks, six weeks, three months, and six months postoperatively. Standardized postoperative photographs were taken during both the early (6 weeks to 6 months) and late (beyond 6 months) follow-up periods. The aesthetic angles and NOSE scores recorded preoperatively were compared with postoperative values. Comparative analyses were also performed between the open and closed approach groups. Postoperative complications and overall patient satisfaction were assessed. Final feedback was collected from both the patients and their attendants regarding functional and aesthetic outcomes.

Statistical analysis

Categorical data were expressed as numbers and percentages, while continuous variables were presented as mean ± SD. A paired t-test was used to evaluate the significance of differences between pre- and postoperative values.

## Results

Demographics

The study included a total of n=30 patients, with n=17 males (56.67%) and n=13 females (43.33%), giving a male-to-female ratio of approximately 1.3:1. The age range of the patients was 10 to 55 years. Equal numbers of patients (n=15 each) underwent rhinoplasty through the open (external) and closed (endonasal) techniques (Table 1).

**Table 1:** Patient demographic characteristics.

Parameter	Value
Total patients	30
Male	17 (56.67%)
Female	13 (43.33%)
Male-to-female ratio	1.3:1
Age range	10-55 years
Open rhinoplasty	15 patients
Closed rhinoplasty	15 patients

Indications and etiology of deformity

Of the n=30 patients, 70% (n=21) presented with both external nasal deformity and nasal obstruction, while the remaining 30% (n=9) presented with external deformity alone. The etiological distribution revealed that 40% (n=12) of cases were post-traumatic, 33.33% (n=10) were developmental, 23.33% (n=7) were congenital, and one case (3.33%, n=1) was post-operative (iatrogenic). The anatomical involvement showed that 46.67% (n=14) had deformities affecting both the upper two-thirds and lower one-third of the nose, while 33.33% (n=10) had deformities limited to the lower third and 20% (n=6) to the upper two-thirds.

In the upper two-thirds, the most frequent deformities included dorsal humps (80%, n=6), dorsal deviation (60%, n=12), wide bony vaults (25%, n=5), and lateral wall depression (20%, n=4). In the lower one-third, common findings included reduced columellar support (41.67%, n=10), under-projected tips (20.83%, n=5), and caudal septal deviations (20.83%, n=5) (Table 2).

**Table 2:** Clinical profile and anatomical distribution of nasal deformities (n = 30).

Category	Subcategory	No. of Patients	Percentage (%)
Indications for surgery	External nasal deformity only	9	30
	External deformity with nasal obstruction	21	70
	Total	30	100
Etiology of deformity	Post-traumatic	12	40
	Developmental	10	33.33
	Congenital	7	23.33
	Post-operative (iatrogenic)	1	3.33
	Total	30	100
Anatomical region involved	Both upper two-thirds and lower one-third	14	46.67
	Lower one-third only	10	33.33
	Upper two-thirds only	6	20
	Total	30	100
Upper two-thirds deformities (n = 20)	Dorsal hump	16	80
	Dorsal deviation	12	60
	Wide bony vault	5	25
	Lateral wall depression	4	20
Lower one-third deformities (n = 24)	Reduced columellar support	10	41.67
	Under-projected tip	5	20.83
	Caudal septal deviation	5	20.83

Functional outcomes (NOSE score)

The functional outcomes were assessed using the NOSE score preoperatively and at >6 months postoperatively. Patients undergoing open rhinoplasty showed a significant improvement, with mean NOSE scores reducing from 36.7 ± 25.26 to 8.3 ± 4.08. Similarly, the closed rhinoplasty group improved from a mean of 38.3 ± 22.09 to 10.0 ± 5.00. Both techniques were effective in improving nasal airflow symptoms, with slightly better mean improvement seen in the open approach group (Table 3).

**Table 3:** Functional outcome: preoperative and postoperative comparison. NOSE: Nasal Obstruction Symptom Evaluation.

Technique	Preoperative NOSE Score (Mean ± SD)	Postoperative NOSE Score (Mean ± SD)
Open approach	36.7 ± 25.26	8.3 ± 4.08
Closed approach	38.3 ± 22.09	10.0 ± 5.00

Aesthetic outcomes

Subjective satisfaction with aesthetic results was assessed at ≥6 months of follow-up. In the open approach group, n=12 (80%) of patients reported being very satisfied, and n=2 (13.3%) were moderately satisfied. In contrast, in the closed group, n=9 (60%) were very satisfied, n=4 (26.67%) were moderately satisfied, and n=2 (13.3%) were unsatisfied (Table 4).

**Table 4:** Satisfactory approach in both groups.

Satisfaction level	Open approach % (n)	Closed approach % (n)
Very satisfied	80.00 (12)	60.00 (9)
Moderately satisfied	13.33 (2)	26.67 (4)
Unsatisfied	0.00 (0)	13.33 (2)

Complications

Intraoperative and postoperative complications were observed in both the open and closed rhinoplasty groups, with the open approach showing a lower incidence of persistent nasal obstruction and aesthetic dissatisfaction. In terms of intraoperative events, alar cartilage injury was noted in one patient in the open group, while septal flap tears occurred in two patients in the closed group. Postoperatively, complications in the open group included epistaxis in n=2 (13.3%) cases, mild persistence of nasal deviation in another n=2 (13.3%), and columellar retraction in n=1 (6.67%). In comparison, the closed group had a higher complication rate, with n=3 (20%) experiencing persistent nasal obstruction, n=4 (26.67%) presenting with persistent nasal deviation, and n=2 (13.3%) developing intranasal synechiae. These findings suggest that the open approach may be associated with a more favorable postoperative course in terms of functional and aesthetic outcomes. The open approach demonstrated fewer complications and higher subjective satisfaction, though it required more extensive dissection and a longer operative time (Table 5).

**Table 5:** Complications in both open and closed approaches.

Complication	Open (n = 15)	Closed (n = 15)
Intraoperative septal flap tear	0 (0%)	2 (13.33%)
Alar cartilage injury	1 (6.67%)	0 (0%)
Postoperative epistaxis	2 (13.33%)	1 (6.67%)
Synechiae formation	0 (0%)	2 (13.33%)
Persistent nasal obstruction	0 (0%)	3 (20.00%)
Persistent nasal deviation	2 (13.33%)	4 (26.67%)
Unsatisfactory aesthetic result	1 (6.67%)	4 (26.67%)

## Discussion

This study was conducted to evaluate and compare the outcomes of open and closed rhinoplasty techniques in patients presenting with external nasal deformities, with or without nasal obstruction. A total of 30 patients were included and equally divided between the two surgical approaches. The results of this study provide insights into the demographic distribution, etiological patterns, anatomical deformities, surgical outcomes, and complications associated with both techniques.

The majority of patients were male (56.67%), which contrasts with global trends showing a predominance of females seeking aesthetic nasal correction. Studies by Foda HM, Teichgraeber JF et al., and Sinha V et al. report higher female-to-male ratios, while the findings of Dhingra PL et al., which reflect a predominantly male cohort, align with our results [15,16]. This demographic trend may be explained by a higher incidence of trauma-related nasal deformities in males due to greater involvement in outdoor activities and sociocultural factors that limit female access to elective surgeries in this region [17]. Most patients in this study (70%) presented with both functional (nasal obstruction) and aesthetic complaints. This observation is consistent with findings by Sinha V et al., Dhingra PL et al., and Friedman GD and Gruber RP, who reported similar proportions of patients with combined concerns. These results reinforce the need for rhinoplasty to address both appearance and nasal airflow, necessitating careful preoperative functional assessment [17-19].

Post-traumatic deformities were the most common etiology in our cohort (40%), followed by developmental (33.33%), congenital (23.33%), and post-operative (3.33%) causes. These findings mirror the observations made by Dziewulski P et al. and Nunes FB et al., further supporting trauma as the leading contributor to structural nasal issues. Anatomically, nearly half the patients (46.67%) had complex deformities involving both the upper and lower thirds of the nose, highlighting the need for versatile surgical planning [20,21]. Analysis of the upper nasal third showed that dorsal hump (76.67%) and dorsal deviation (40%) were the most frequent deformities. These findings closely align with Foda HM’s study, where dorsal humps and deviations were also dominant. In contrast, deformities in the lower nasal third, such as reduced columellar support (33.33%) and under-projected tips (16.67%), were less prevalent than reported in other series, indicating regional or population-based variability [15].

Septal deviations were present in 70% of patients, with C-shaped cephalocaudal deviation being the most common. This contrasts with findings where septal tilt was more frequent, reflecting either racial anatomical differences or differences in classification methodology [22]. The role of septal correction in rhinoplasty is further supported by Beekhuis’ principle that “as the septum goes, so goes the nose,” emphasizing the importance of functional realignment. Autologous cartilage grafting was employed in 15 patients, with septal cartilage being the most commonly used graft material (86.67%). Techniques such as spreader grafts and columellar struts were used strategically, particularly in the open approach. These practices were consistent with those reported by Berger CA et al. and Gendeh BS, supporting autologous cartilage as a safe and effective option in nasal reconstruction [23,24]. In terms of complications, the closed approach was associated with a higher incidence of synechiae formation, persistent deviation, and postoperative nasal obstruction. Conversely, the open approach had fewer complications and better subjective outcomes. Only one patient in the open group reported columellar scar visibility, which was deemed acceptable. These results support existing literature emphasizing that, while the open approach may involve more extensive dissection, it allows for precise correction and fewer secondary issues [25].

Aesthetic satisfaction was higher in the open approach group, with 80% of patients being very satisfied compared to 60% in the closed group. However, two patients in the closed group reported no functional benefit. These findings are comparable to those reported by Yeung A et al., Rhee JS et al., and Sinha V et al., confirming rhinoplasty's effectiveness in relieving nasal obstruction while improving facial harmony [26,27]. Importantly, aesthetic nasal angle analysis revealed that in female patients undergoing open rhinoplasty, both the nasofrontal and nasolabial angles showed improvement. These findings suggest that aesthetic improvements through open rhinoplasty may be more perceptible or measurable in female patients, possibly due to higher baseline aesthetic expectations or anatomical subtleties. Similar observations were reported by Berger CA et al. and Szychta P et al., who found more consistent angle improvements in females [23,28].

Normative nasal angle values were also established in a healthy Kashmiri control group. The mean nasofrontal, nasofacial, and nasolabial angles were found to be within acceptable aesthetic ranges, though they varied slightly from ideal Caucasian parameters reported in studies by Leong SC et al. This reinforces the importance of considering ethnic variability in aesthetic planning [29].

Limitations

The primary limitations of this study include its relatively small sample size of 30 patients, which may affect the statistical power and generalizability of the results to larger or more diverse populations. The study was conducted at a single center, limiting external validity. Additionally, the follow-up period ranged from 7 to 12 months, which may not be sufficient to assess long-term aesthetic stability and functional outcomes.

## Conclusions

This study demonstrates that both open and closed rhinoplasty techniques are effective in correcting external nasal deformities and improving nasal function. However, the open approach provided better surgical exposure, allowing for more precise anatomical corrections, especially in cases involving complex deformities. It was associated with higher patient satisfaction, fewer complications, and statistically significant aesthetic improvements in female patients, particularly in the nasolabial and nasofrontal angles. The closed approach, while less invasive, showed a slightly higher incidence of postoperative complications with residual deformities. Functional outcomes, as measured by NOSE scores, improved significantly in both groups, underscoring the importance of septal correction in rhinoplasty. Ultimately, the choice of surgical approach should be tailored to the individual patient’s anatomical needs, functional complaints, and aesthetic expectations, with consideration of the surgeon’s experience and the complexity of the deformity.
